# Outcomes of endoscopic transsphenoidal surgery for Cushing's disease

**DOI:** 10.1186/s12902-021-00679-9

**Published:** 2021-03-03

**Authors:** Zarina Brady, Aoife Garrahy, Claire Carthy, Michael W. O’Reilly, Christopher J. Thompson, Mark Sherlock, Amar Agha, Mohsen Javadpour

**Affiliations:** 1 Medical University of Varna, Varna, Bulgaria; 2 Department of Neurosurgery, Beaumont Hospital, Dublin, Ireland; 3 Academic Department of Endocrinology, Beaumont Hospital, Dublin, Ireland; 4 Royal College of Surgeons in Ireland, Dublin, Ireland

**Keywords:** Cushing’s disease, Endoscopic, Transsphenoidal surgery, Pituitary adenoma

## Abstract

**Background:**

Transsphenoidal surgery (TSS) to resect an adrenocorticotropic hormone (ACTH)-secreting pituitary adenoma is the first-line treatment for Cushing’s disease (CD), with increasing usage of endoscopic transsphenoidal (ETSS) technique. The aim of this study was to assess remission rates and postoperative complications following ETSS for CD.

**Methods:**

A retrospective analysis of a prospective single-surgeon database of consecutive patients with CD who underwent ETSS between January 2012–February 2020. Post-operative remission was defined, according to Endocrine Society Guidelines, as a morning serum cortisol < 138 nmol/L within 7 days of surgery, with improvement in clinical features of hypercortisolism. A strict cut-off of < 50 nmol/L at day 3 post-op was also applied, to allow early identification of remission.

**Results:**

A single surgeon (MJ) performed 43 ETSS in 39 patients. Pre-operative MRI localised an adenoma in 22 (56%) patients; 18 microadenoma and 4 macroadenoma (2 with cavernous sinus invasion). IPSS was carried out in 33 (85%) patients. The remission rates for initial surgery were 87% using standard criteria, 58% using the strict criteria (day 3 cortisol < 50 nmol/L). Three patients had an early repeat ETSS for persistent disease (day 3 cortisol 306-555 nmol/L). When the outcome of repeat early ETSS was included, the remission rate was 92% (36/39) overall. Remission rate was 94% (33/35) when patients with macroadenomas were excluded. There were no cases of CSF leakage, meningitis, vascular injury or visual deterioration. Transient and permanent diabetes insipidus occurred in 33 and 23% following first ETSS, respectively. There was one case of recurrence of CD during the follow-up period of 24 (4–79) months.

**Conclusion:**

Endoscopic transsphenoidal surgery produces satisfactory remission rates for the primary treatment of CD, with higher remission rates for microadenomas. A longer follow-up period is required to assess recurrence rates. Patients should be counselled regarding risk of postoperative diabetes insipidus.

## Introduction

With an estimated annual incidence of 1.7 per million [1], Cushing’s disease is rare. Untreated, it poses serious complications including osteoporosis, hypertension, dyslipidaemia, insulin resistance, and hypercoagulability [2] and is associated with a 4.8 fold increase in mortality rate [3–5]. Patients who are in remission from CD have a mortality rate which decreases towards (although not reaching) that of the general population [6]. Endoscopic transsphenoidal surgery (ETSS) offers patients potential remission from Cushing’s disease, although long term surveillance is required as recurrence rates range from 5 to 22%% [7–12].

Since the first report in 1997 [13], the selective removal of an adrenocorticotropic hormone (ACTH)-secreting pituitary adenoma by endoscopic transsphenoidal surgery has gained popularity as the first line treatment for Cushing’s disease. The primary goal of ETSS treatment in Cushing’s disease is to produce disease remission and to provide long-term control, while minimising complications. Remission rates are dependent on tumour size, preoperative MRI, cavernous sinus invasion, intraoperative visualisation of the tumour and pre- and postoperative ACTH and cortisol concentration [11]. Several studies also report pituitary neurosurgeon experience as a major factor for operative success [2, 14, 15].

Reported remission and recurrence rates after TSS for CD vary widely according to the criteria utilised to define remission [11], and in some studies due to limited patient numbers or short follow-up periods. Indeed, there is no clear consensus on how best to define post-operative remission; an early morning serum cortisol concentration < 138 nmol/L (5μg/dl) within 7 days of TSS is quoted in the 2015 Endocrine Society Clinical Practice Guideline as indicative of remission [16]. A more strict day 3 cut-off of 50 nmol/L (1.8 μg/dl) has been reported in paediatric studies [17], and also included in the Endocrine Society Guideline [16]; the literature suggests this cut-off is associated with remission, and a low recurrence rate of approximately 10% at 10 years [14]. The main objective of this study was to assess the outcomes of endoscopic transsphenoidal surgery for Cushing’s disease in a tertiary pituitary centre; remission using two widely accepted criteria [16], recurrence and postoperative complications.

## Methods

### Study design

This is a retrospective analysis of a prospectively-maintained database of patients operated on by a single neurosurgeon (MJ), via image-guided endoscopic transsphenoidal approach for Cushing’s disease. Patient data was gathered over 8 years (January 2012 to February 2020) and identified from the institution’s prospective database. Clinical and biochemical data during the follow-up period was reviewed. Approval was granted by the Hospital Audit Committee.

### Study population

Patients were screened for Cushing’s syndrome by the presence of typical clinical features, together with failure to adequately suppress cortisol to < 50 nmol/L following overnight dexamethasone suppression test (ONDST) and/or elevated late night salivary cortisol (LNSF) concentration and/or elevated 24 h urinary free cortisol measurements. As per standard guidelines, Cushing’s disease was diagnosed on the basis of elevated serum ACTH measurements, along with confirmatory hormone responses to peripheral corticotropin releasing hormone (CRH) test and inferior petrosal sinus sampling (IPSS). Patients with previous TSS prior to the study period were excluded.

### Surgical procedure

A single neurosurgeon subspecialising in endoscopic pituitary and anterior skull base surgery, M.J, carried out all ETSS surgical procedures. The surgical technique has been described in detail in publications by Cappabianca et al. (1998, 1999) and Jho et al. (1997, 2000, 2001) [13, 18–21]. In summary, the procedure consists of a binostril endoscopic transsphenoidal approach. A selective adenomectomy was performed on patients with adenomas noted on pre-operative MRI. In cases of negative pre-operative MRI, exploration of the pituitary gland was performed. To confirm the diagnosis of ACTH-secreting adenoma or hyperplasia, all specimens removed underwent histopathological and immunohistochemical staining for pituitary hormones.

### Postoperative assessment

Patients received empiric oral hydrocortisone on day 1 and on the morning of day 2 post-operatively, prior to assessment of 0800 h serum cortisol on day 3. A blood sample for serum cortisol was drawn at 0800 h on the morning of day 3, if clinically stable, prior to administration of hydrocortisone. The Endocrine Society Clinical Practice Guideline define post-operative biochemical remission as morning serum cortisol < 138 nmol/L (5μg/dl) within 7 days postoperatively [16], ‘standard criteria’. In our institution, we also apply a biochemical cut-off of < 50 nmol/L (1.8 μg/dl) at day 3 postoperatively to allow early indication of biochemical remission, ‘strict criteria’. If serum cortisol on day 3 is 50–138 nmol/L, serial measurements are taken daily to determine if cortisol will fall further, and assessment for improvement/resolution of clinical sequalae of hypercortisolaemia made (such as improvement in blood pressure or glycaemic control), before repeat endoscopic transsphenoidal surgery is considered.

Transient cranial diabetes insipidus (DI) was defined as the development of hypotonic polyuria postoperatively requiring at least one dose of desmopressin [22], which resolved prior to discharge. Permanent DI was confirmed by water deprivation test according to standard criteria [23]. Thyroid stimulating hormone (TSH) deficiency was defined by low fT4 with either low or inappropriately normal TSH. Growth hormone (GH) deficiency was confirmed using either Insulin Tolerance Test or Glucagon Stimulation Test [24]. Gonadotrophin deficiency was defined in premenopausal women as amenorrhoea with inappropriately low FSH and LH concentration, and in postmenopausal patients as inappropriately low FSH and LH concentration.

Recovery of hypothalamic-pituitary-adrenal axis was assessed by short synacthen (250 μg) test or insulin tolerance test 3 months post-operatively, and every 3–6 months thereafter in cases of initial fail or borderline result. Patients were assessed annually for recurrence of Cushing’s disease, recurrence was defined by failure to suppress cortisol to < 50 nmol/L following an 1 mg overnight dexamethasone suppression test, an elevated late night salivary cortisol (LNSF) or urinary free cortisol (UFC) in patients no longer taking hydrocortisone.

### Laboratory analysis

Prior to 2019, serum cortisol was measured using a chemiluminescent immunoassay with the Beckman Coulter UniCel Dxl 800. Intra-assay CV for serum cortisol was 8.3, 5 and 4.6% at concentrations of 76, 438 and 865 nmol/L, respectively. From January 2019 onwards, serum cortisol was measured using Elecsys® Cortisol II assay on the Roche Cobas e801; intra-assay precision for serum cortisol was 1.2, 1.1 and 1.6% at concentrations of 31.8, 273 and 788 nmol/L, respectively.

### Statistics

Data are expressed as median (range) and number (%). The Fishers Exact test was used to compare categorical variables between groups. All *p*-values were considered statistically significant at a level < 0.05. Statistical analysis was performed using GraphPad Prism 8 statistical software (GraphPad Software, La Jolla, California, USA).

## Results

### Demographics

Forty-three endoscopic transsphenoidal procedures were performed in 39 patients. Demographics are summarised in Table 1. Median (range) age was 37 years (8–75), 30 were female. Median (range) duration of symptoms was 24 months (6–144), 72% (28/39) had hypertension, and 28% (11/39) had type 2 diabetes.

**Table 1:** Summary of demographics and post-operative outcomes

Number of patients undergoing ETSS	39
Number of ETSS procedures	43
Age	37 (8–75)
Female	30 (77%)
Tumour visible on pre-op MRI *Microadenoma* *Macroadenoma*	22 (56%) 18 4
Pre-operative IPSS	33 (85%)
Postoperative remission, initial ETSS	
*Standard criteria*^a^	34 (87%)
*Strict criteria*^b^	22 (58%)
Postoperative remission, including 3 patients with early repeat ETSS	
*Standard criteria*^a^	36 (92%)
*Strict criteria*^b^	23 (61%)
Postoperative hypopituitarism	
*Transient diabetes insipidus*	13 (33%)
*Permanent diabetes insipidus*	9 (23%)
*TSH deficiency*	5 (13%)
*Gonadotrophin deficiency*	4 (10%)
Recurrence	1

Data expressed as median (range) and number (percentage).

^a^ 0800 h serum cortisol < 138 nmol/l within seven days of operation and improvement in clinical features of hypercortisolism

^b^ Day 3 0800 h serum cortisol concentration < 50 nmol/L. Day 3 cortisol was not measured in one patient due to intercurrent illness requiring treatment with intravenous glucocorticoids

### Preoperative imaging and IPSS

Pre-operative MRI localised an adenoma in 22 (56%) patients; 18 microadenoma and 4 macroadenoma (2 with cavernous sinus invasion). No adenoma was identified in 17 patients (44%). IPSS was carried out in 33 (85%) patients.

### Postoperative remission

**Fig. 1:**
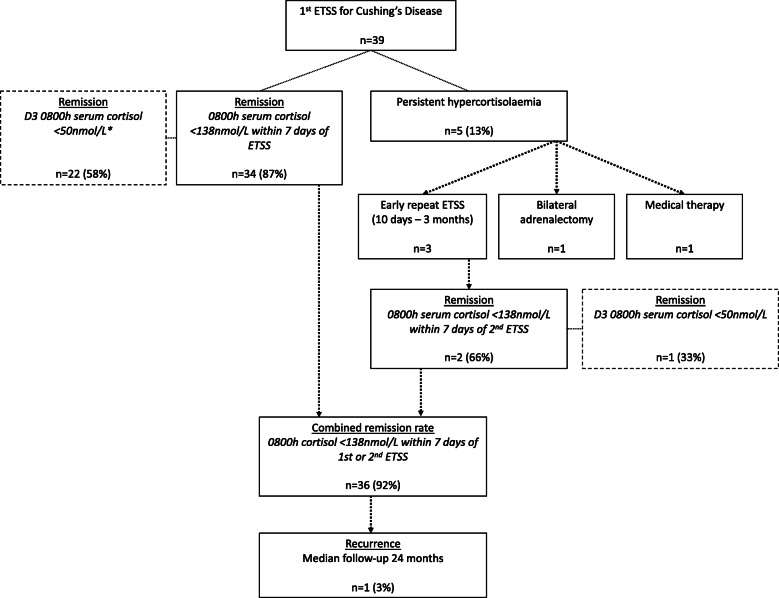
Schema of patients who underwent ETSS. *Day 3 cortisol was not measured in one patient due to intercurrent illness requiring treatment with intravenous glucocorticoids

Post-operative outcomes are summarised in Table 1 and Fig. 1. Using standard criteria (0800 h serum cortisol < 138 nmol/l within 7 days of operation and improvement in clinical features of hypercortisolism), postoperative remission rates for initial surgery were 87% (34/39) for the entire group and 89% (31/35) when patients with macroadenomas were excluded, Fig. 1. Three patients had an early repeat ETSS for persistent disease; day 3 serum cortisol ranged from 306 to 555 nmol/L and interval to repeat ETSS from 10 days–3 months. When the outcome of early repeat ETSS was factored in, overall remission rate was 92% (36/39) overall, and 94% (33/35) when patients with macroadenomas were excluded.

Using strict criteria of early remission (day 3 serum cortisol concentration < 50 nmol/L), postoperative remission rates were 58% (22/38) overall, and 62% (21/34) excluding macroadenomas. Including the three patients with early repeat ETSS, remission rate was 61% (23/38) overall, and 65% excluding macroadenomas (22/34). Day 3 cortisol was not measured in one patient due to intercurrent illness requiring treatment with intravenous glucocorticoids.

Eleven patients (28%) had a cortisol measurement between 50 and 138 nmol/L on day 3, seven of whom had received metyrapone therapy prior to ETSS. Six patients had serial measurements of 0800 h cortisol up to a maximum follow-up of 14 days post-op, serum cortisol concentration fell after day 3 in all six patients. Ten (91%) were glucocorticoid-dependent at 3 months based on synacthen/ITT; 0800 h cortisol had fallen to < 50 nmol/L in six patients.

### Predictors of remission

No statistical difference was found in the rates of remission in those patients with or without tumour target on preoperative MRI, using either strict criteria for remission (12/21 target vs 10/17 no target, *p* > 0.99) or standard criteria (19/22 target vs 15/17 no target, p > 0.99). Similar results were found when the four patients with macroadenoma were excluded.

### Persistent disease

Five patients (13%) had persistent hypercortisolaemia after the initial endoscopic transsphenoidal surgery (Table 2). Three patients underwent a repeat early endoscopic transsphenoidal surgery, Fig. 1. Remission rate after repeat early ETSS was 67% (2/3) using standard criteria, and 33% (1/3), using the strict criteria. Of the patients with persistent disease following repeat ETSS, one received radiosurgery, while the other has been commenced on medical therapy, with a view to refer for radiotherapy.

**Table 2:** Outcome of five patients with persistent hypercortisolaemia after initial ETSS

	Pre-operative MRI	D3 0800h serum cortisol (nmol/L) 1st ETSS	Subsequent management
1	Microadenoma	555	Second ETSS – in remission, D3 cortisol 41 nmol/L.
2	Microadenoma	306	Second ETSS – persistent mild hypercortisolemia, D3 cortisol 80 nmol/L.
3	Nil visible	386	Second ETSS – persistent disease, D3 cortisol 379 nmol/L. Medical therapy with cabergoline and stereotactic radiosurgery.
4	Nil visible	779	Bilateral adrenalectomy
5	Macroadenoma with cavernous sinus invasion	497	Medical therapy with metyrapone. Fractionated pituitary radiotherapy

### Postoperative complications

The rate of transient diabetes insipidus after first ETSS was 33% (13/39), while permanent diabetes insipidus occurred in 23% (9/39). Postoperatively, there were five cases of new thyroid stimulating hormone deficiency (13%) and four cases of gonadotrophin deficiency (10%) (in pre-menopausal females). There were no cases of postoperative CSF leak, no cases of meningitis and no visual complications. There were no other complications.

### Recurrence

No patients were lost to follow-up. Over a median (range) duration of follow-up of 24 (4–79) months, one patient had recurrence of Cushing’s disease. Pre-operative MRI had shown a macroadenoma; serum cortisol on day 3 after the initial ETSS was 71 nmol/L, which fulfilled standard criteria for remission, but not the more strict criteria. The patient underwent a second ETSS 13 months later. No tumour was visible intra-operatively so no tissue was removed, day 3 serum cortisol concentration was 308 nmol/L and the patient was commenced on a trial of metyrapone.

### Recovery of the hypothalamic-pituitary-adrenal axis

Recovery of the hypothalamic-pituitary-adrenal axis occurred in nine patients (27%), at median 13 (3–27) months post-operatively. There was no statistical difference in rates of recovery of HPA axis in patients with day 3 cortisol < 50 nmol/l, and those who only passed standard criteria for remission (< 138 nmol/l) [7/20 (follow-up 25 (3–59) months) versus 2/11 (follow-up 16 (3–79) months) respectively, *p* = 0.43]. One patient died 5 weeks post-operatively; post-mortem revealed bilateral haemorrhagic adrenal necrosis.

## Discussion

Reported remission rates following ETSS in patients with Cushing’s disease (CD) vary widely, predominantly due to differences in criteria used to define remission [11]. There is no uniform consensus on the criteria used to define ‘remission’, with institutions using a combination of biochemical and clinical criteria; this makes comparing surgical outcome studies challenging. The normal corticotroph cells of the pituitary gland are suppressed due to sustained hypercortisolaemia, therefore following successful removal of the ACTH-secreting adenoma, serum ACTH and cortisol concentrations should fall postoperatively. A morning serum cortisol concentration < 138 nmol/L (5 μg/dl) within 7 days of ETSS is usually indicative of remission, and this biochemical cut-off is quoted in the Endocrine Society Clinical Practice Guideline [16], and many surgical outcome studies [8, 11, 25]. Other studies have applied a more strict serum cortisol cut-off of < 50 nmol/L (1.8 μg/L) at day 3 postoperatively to allow early indication of biochemical remission [10, 11, 26–28]; the literature suggests this cutoff is associated with remission, and a low recurrence rate of approximately 10% at 10 years [14]. Our practice is to apply this latter approach; if serum cortisol on day 3 is 50–138 nmol/L, serial measurements are taken daily to determine if cortisol will fall further, and assessment for improvement/resolution of clinical signs of hypercortisolaemia made, before repeat endoscopic transsphenoidal surgery is considered. It is important to ensure that serum cortisol has reached a nadir, before further intervention is considered.

In this single-centre single-surgeon study, we report two very different remission rates using these two widely accepted criteria. Our remission rate, including those patients who had an early second ETSS, using standard guidelines, is 92%, on par with other larger studies [7, 8, 11, 25, 29]. When patients with corticotroph macroadenomas were excluded, the remission rate was even higher at 94%. In comparison, when we applied the more strict criteria of day 3 cortisol < 50 nmol/L, the remission rate was considerably lower at 61%. This criteria is in place in our institution so that we can safely identify patients who have early signs of remission to facilitate discharge on day 3 post-operatively; however reporting these rates in isolation lead to a misleadingly low remission rate compared to the more lenient criteria proposed by the Endocrine Society [16].

Evidence has suggested that higher day 3 cortisol concentration is associated with greater risk of recurrence of CD. A recent retrospective cohort analysis of 81 ETSS for CD by Mayberg et al. reported significantly higher recurrence rates in patients with post-operative cortisol nadir between 58 and 149 nmol/L (2.1–5.4 μg/dL) compared with those with cortisol < 55 nmol/L (2 μg/dL) (33% vs 6%, *p* = 0.01) [30]. Recurrence of CD was low in our series at 3%, and occurred in a patient with a corticotroph macroadenoma, which have been shown to be associated with higher rates of recurrence [31]. On post-operative assessment, serum cortisol fell between the two criteria for remission and if remission was strictly defined as a day 3 cortisol < 50 nmol/L, then this patient had in fact persistent hypercortisolaemia. This case highlights the difficulty when comparing studies reporting ETSS outcomes in CD – the distinction between persistent post-operative hypercortisolism and early recurrence of CD is not always clear-cut, and is dictated by the local protocol.

Whilst our recurrence data are encouraging in comparison to other reports on CD recurrence, which published rates of up to 22% [11], longer term follow-up is necessary before recurrence rates can be accurately defined. The criteria used to define long term recurrence of CD also varies widely in the literature; a large systematic review (*n* = 6400) by Petersenn et al. (2015) reported decreased recurrence rates when studies used UFC with ONDST vs. UFC only, and UFC with morning serum cortisol vs. UFC only [11]. This highlights the requirement for standardization of remission and recurrence criteria, for consistency in clinical practice and in the literature.

The post-operative surgical complication rate in our series was very low, with no cases of CSF leak, vascular injury or visual compromise. Other published case series have reported incidence rates for CSF leakage and meningitis of 0–7.2% and 0–7.9% [2, 12, 32, 33] respectively. Postoperative meningitis is strongly associated with CSF leakage [34]. Some studies suggest that the endoscopic approach results in higher rates of carotid artery injury compared with the microscopic approach, which could be attributed to the nature of the extended lateral approach [35]. However, in this series of 43 ETSS, we report no cases of surgical related carotid artery injury, similar to other studies reporting 0% serious morbidity or mortality due to carotid artery injury [33, 36]. Finally, postoperative visual disturbance is a major concern, as it can be life changing for patients. Factors linked with visual complications include tumour size, patient age and any pre-existing visual conditions [37–39]. Visual deterioration after TSS for Cushing’s disease has been reported to occur in some large case series at rates of 1.9% [32] and 0.86% [12]. There were no cases of postoperative visual disturbance in our series.

While the surgical complication rate was low, our endocrine complication rate was higher than that reported in other studies, particularly the rate of DI. Transient DI occurred in 33% of cases, and permanent DI in 23%. These relatively high rates of transient DI may be due to the diagnostic criteria used in our protocol; we defined transient post-operative DI as one episode of hypotonic polyuria in the setting of normal or elevated plasma sodium concentration, requiring at least one dose of desmopressin. In contrast, some studies discount any polyuria which lasts less than 2 days [10], while others require the documentation of hypernatremia for the diagnosis of DI [40]. These more stringent criteria will not capture cases of mild transient DI; therefore it is not surprising that the rates of transient DI reported in a 2018 meta-analysis were lower than that in our study, 11.3% [29]. The rates of permanent DI in our study merits particular attention. TSS for CD has been shown to be associated with a higher risk of post-operative DI [41, 42]. It may be that a more aggressive surgical approach resulted in high remission rates, but at a cost of higher rates of DI. All patients are reviewed post-operatively in the National Pituitary Centre, where there is a low threshold for water deprivation testing and/or 3% saline testing. We did not routinely re-test patients for resolution of DI after their initial water deprivation test at 3 months, and it is possible that some cases subsequently resolved after 3 months [41, 43]. Regardless, the rate reported in this study is significant, and emphasises the importance of counselling the patient about the risk of DI long-term.

### Strengths and limitations

The reporting of two remission rates based on widely accepted criteria is a strength of this study, and allows for direct comparison of our outcomes with other studies. All ETSS were performed by a single pituitary surgeon; while this removes bias from surgeon experience, the disadvantage of this is that the sample size is relatively low. Furthermore, because we included patients who were recently operated on to maximise numbers for analysis of surgical complications, the follow-up period is relatively short. A longer follow-up is required to comment accurately on recurrence of CD. We did not have full ascertainment of longitudinal post-operative data including dexamethasone suppression tests, and this has highlighted the need for protocolised follow-up to allow for consistency when reporting our results.

## Conclusion

Endoscopic transsphenoidal surgery in patients with Cushing’s disease offers excellent remission rates and low morbidity. Remission rates are much higher when standard criteria [morning serum cortisol < 138 nmol/L (5μg/dl) within 7 days postoperatively] are used compared with day 3 cortisol < 50 nmol/l. Higher remission rates were found for patients with microadenomas. Patients should be counselled regarding risk of post-operative endocrine deficiencies, in particular permanent diabetes insipidus. Longer follow-up is required to accurately assess recurrence rates.

## Data Availability

The data that support the findings of this study are not publicly available due to restrictions by General Data Protection Regulation (GDPR), but are available from the corresponding author on reasonable request.

## Abbreviations

TSS: Transsphenoidal surgery
ACTH: Adrenocorticotropic hormone
CD: Cushing’s disease
ETSS: Endoscopic transsphenoidal surgery
ONDST: Overnight dexamethasone suppression test
LNSF: Late night salivary cortisol
CRH: Corticotropin releasing hormone
IPSS: Inferior petrosal sinus sampling
DI: Diabetes insipidus
TSH: Thyroid stimulating hormone
GH: Growth hormone
UFC: Urinary free cortisol
